# Dataset of fluoride concentration and health risk assessment in drinking water in the Saveh City of Markazi Province, Iran

**DOI:** 10.1016/j.dib.2023.109466

**Published:** 2023-08-02

**Authors:** Hamid Reza Tashauoei, Mokhtar Mahdavi, Amir Hossein Mahvi, Ali Fatehizadeh

**Affiliations:** a Department of Environmental Health Engineering, Faculty of Public Health and Biomedical Engineering, Tehran Medical Sciences, Islamic Azad University, Tehran, Iran; b Assistant Professor of Environmental Health Engineering Department, Saveh University of Medical Sciences, Saveh, Iran; c Social Determinants of Health Research Center, Saveh University of Medical Sciences, Saveh, Iran; d Department of Environmental Health Engineering, School of Public Health, Tehran University of Medical Sciences, Tehran, Iran; e Center for Solid Waste Research, Institute for Environmental Research, Tehran University of Medical Sciences, Tehran, Iran; g Department of Environmental Health Engineering, School of Health, Isfahan University of Medical Sciences, Isfahan; h Environment Research Center, Research Institute for Primordial Prevention of Non-Communicable Disease, Isfahan University of Medical Sciences, Isfahan, Iran

**Keywords:** Fluoride, Drinking water, Health Risk assessment, Saveh

## Abstract

This study aims at analyzing fluoride levels in water sources and drinking water in Saveh City, with a focus on health risks assessment. Excessive fluoride concentrations (above 2 to 4 mg/L) can lead to skeletal issues, dental fluorosis, and brain damage, while low concentrations (below 0.7-1.5 mg/L depending on temperature) can harm tooth health and strength. For drinking water consumptions, centralized and decentralized desalination units were utilized from, Saveh's brackish water. In this research study a total of 63 samples were collected randomly from underground and surface water sources, distribution networks, and desalination units during both Winter and Summer seasons. Fluoride analysis was conducted using the spectrophotometric method with the DR6000 device and SPADNS reagent. The results indicated that during winter, average fluoride concentrations in underground water, water treatment plant output, distribution network, centralized desalination unit output, and decentralized desalination unit output were 0.67, 0.64, 0.62, 0.064, and 0.07 mg/L, respectively. In summer, the average concentrations were 0.79, 0.75, 0.71, 0.04, and 0.07 mg/L, respectively. For desalinated water produced by centralized desalination units during the summer season, the Estimated Daily Intake (EDI) values for fluoride in different age groups, including infants, children, teenagers, and adults, were found to be 0.0003, 0.0023, 0.0016, and 0.0013 mg/kg, respectively. Health risk assessment data indicated Hazard Quotient (HQ) values for fluoride in these age groups were 0.005, 0.037, 0.026, and 0.02, respectively. Similar values were observed in the winter data. However, it is important to note that the fluoride concentration in Saveh's drinking water is nearly zero, and the absence of fluoride in desalinated drinking water can have a negative impact on dental health. Therefore, it is crucial to address the lack of fluoride in the drinking water of this city.

**Specifications Table**

**Table:** 

Subject	Water quality and risk assessment.
Specific subject area	Water fluoride
Data format	Raw and Analyzed
Type of data	Table and Figure
Data collection	Total samples (63 sample) were collected in a random manner, encompassing both Winter and Summer seasons. These samples were gathered from diverse sources such as underground and surface water sources, distribution networks, and desalinated water generated by both centralized and decentralized desalination units. To analyse the fluoride content, the spectrophotometric method was employed using the DR6000 device and SPADNS reagent.
Data source location	Institution: Saveh University of Medical Sciences City/Town/Region: Saveh, Markazi province Country: Iran
Data accessibility	Repository name: Mendeley data Data identification number: 10.17632/b6cd84zy24.1 Direct URL to data: https://data.mendeley.com/datasets/b6cd84zy24/1.

## Value of the Data

• The aim of this study was to analyze the fluoride concentration in drinking water and evaluate its impact on consumer health according to established standard values.
• The Health Risk Assessment (HRA) and data analysis consistently showed Hazard Quotient (HQ) values below 1 for all samples across various age groups, including infants, children, teenagers, and adults.
• These findings indicate that Saveh City's drinking water has minimal fluoride content due to the use of desalination units. Therefore, it is advisable to explore alternative methods such as using fluoride toothpaste, mouthwash, or incorporating fluoride-rich foods like fish, shrimp, and tea into the diet and alternatively, the transfer of freshwater from neighboring cities or provinces.

## 1 Data Description

The dataset featured in this study is uploaded on Mendeley Data. The dataset is available as an Excel file named “data in brief RFD for fluoride assessment.xlsx” and contains four sheets: Summer, Winter, concentration, and map. The Summer and Winter sheets include calculations for Exposure Levels and Health Risk Assessment. The concentration sheet displays the fluoride levels in Saveh City, while the final sheet provides information on cities within Markazi province. Also, the data are on a repository (https://data.mendeley.com/datasets/b6cd84zy24/1).

**Fig. 1:**
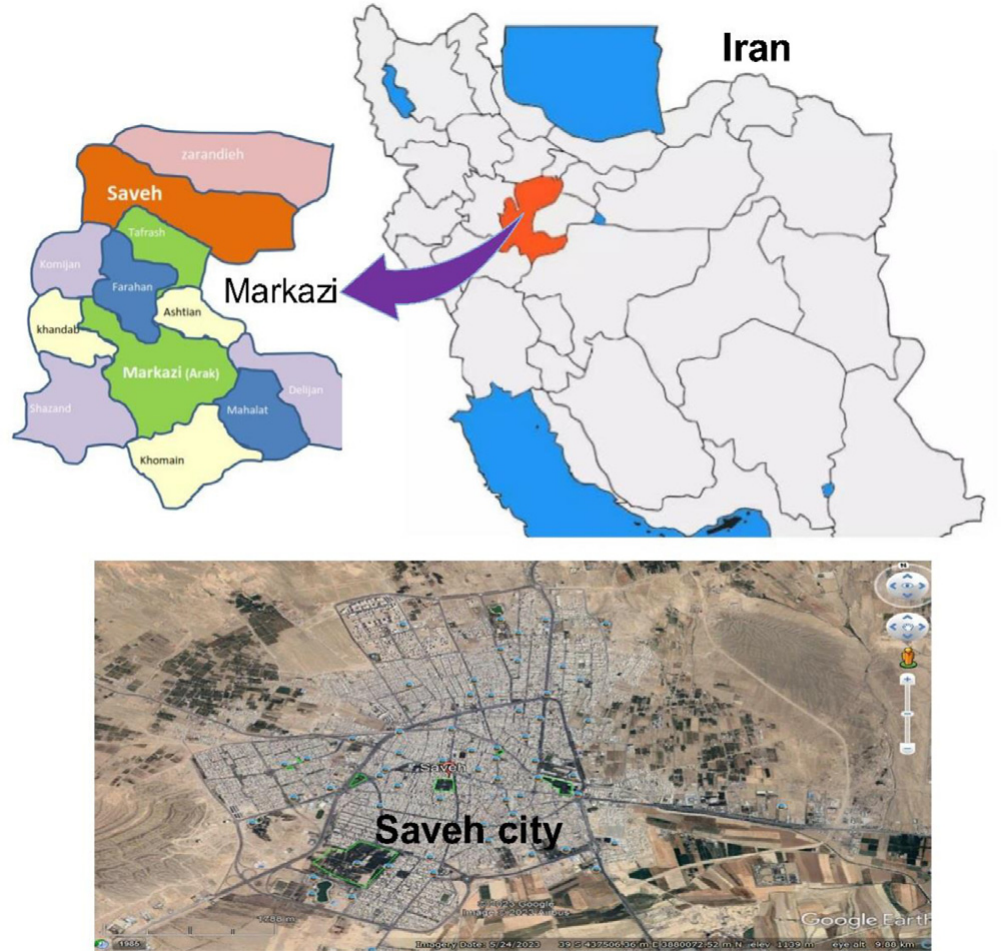
Location map of the study area.

**Locations of City:**Fig. 1 illustrates the locations where water samples were collected in Saveh City, Markazi province, Iran.

**Fluoride concentration in water:**Table 1 presents the fluoride concentrations of various water sources studied in Saveh City during both Winter and Summer seasons. This section show the amount of fluoride measured in water wells, the output of the Saveh water treatment plant, the distribution network, and water treated by both centralized and decentralized desalination units.

**Table 1:** The average concentration of fluoride in all types of water investigated in Saveh City.

Season		Fluoride in well water (mg/L)	Fluoride in WTP output (mg/L)	Fluoride in water network (mg/L)	Fluoride in centralized RO (mg/L)	Fluoride in Household RO (mg/L)	Temperature °C
Winter	Mean	0.67	0.64	0.62	0.064	0.07	11.5
	SD	0.123	0.115	0.11	0.015	0.027	2
Summer	Mean	0.79	0.75	0.71	0.04	0.07	37
	SD	0.07	0.05	0.04	0.014	0.03	2.5
Total	Mean	0.736	0.7	0.668	0.05	0.07	24
	SD	0.11	0.1	0.09	0.01	0.02	2.7

**Fig. 2:**
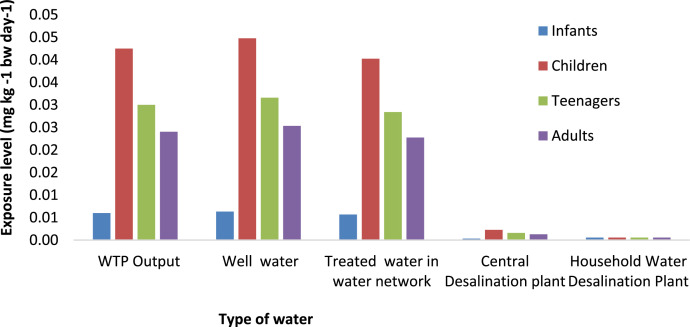
Exposure levels of Fluoride for different type of water in Saveh City over four age groups (infants, children, teenager and adults) in Summer.

**Fig. 3:**
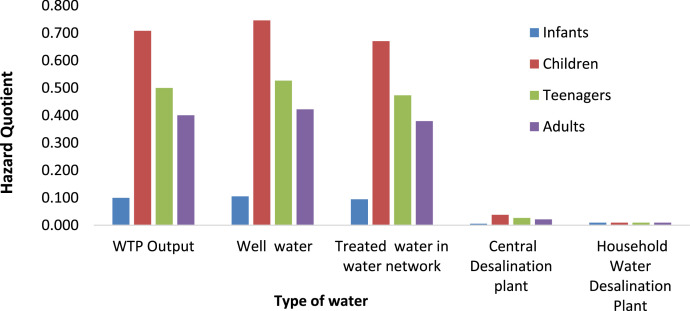
Hazard Quotient value of Fluoride for different type of water in Saveh City over four age groups (infants, children, teenager and adults) in summer.

**Fig. 4:**
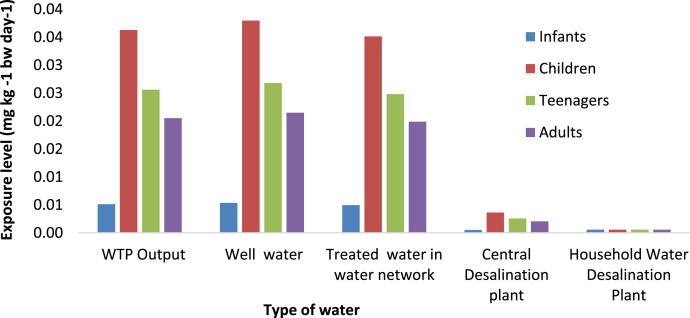
Exposure levels of Fluoride for different type of water in Saveh City over four age groups (infants, children, teenager and adults) in Winter.

**Fig. 5:**
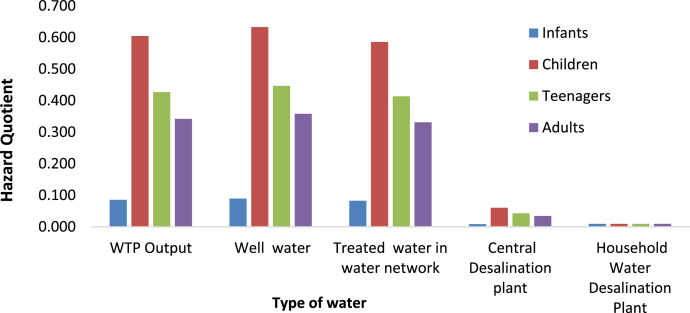
Hazard Quotient value of Fluoride for different type of water in Saveh City over four age groups (infants, children, teenager and adults) in Winter.

**Exposure Levels and Health Risk Assessment:** The exposure levels of fluoride and the HQ values for various age groups (infants, children, teenagers, and adults) are presented in Fig. 2, Fig. 3, Fig. 4, Fig. 5, depicting both summer and winter data.

## 2 Experimental Design, Materials and Methods

### 2.1 Study area

Saveh City, located in the Northern part of Markazi Province in Iran, which is the largest city in the province, covering an area of 8855 square kilometers. It shares borders with Karaj City and Qazvin City to the North and Northwest, Shahryar City by Northeast, Qom City to the Southeast, Tafarsh City to the South, and Hamadan City by West. Saveh City is home to approximately 1000 large factories. The climate in this city is characterized as hot and dry, and its water sources are brackish water. To ensure the provision of safe drinking water, reverse osmosis desalination units are utilized. Therefore, it is crucial to monitor the levels of fluoride, as well as other anions and cations, which can have significant impacts on consumer health. The Universal Transverse Mercator (UTM) coordinates for this city are 441176.84 m E and 3875839.24 m N.

### 2.2 Sample collection and analysis

The sample size for this study was determined using a statistical method. A total of 63 water samples were collected from different locations within Saveh City, including well outlets, the water treatment plant outlet, the water distribution network, and both centralized and decentralized desalination units. These samples were collected during both Summer and Winter seasons. The fluoride concentration in the samples was measured using the SPADNS method with a DR-6000 Hach-Lange spectrophotometer [1].

### 2.3 Exposure and health risk assessment

Human health risk assessment is an important approach used to evaluate the potential impact of hazards, particularly chemicals, on the health of individuals in communities. There are currently some literature reviews on Human Health Risk Assessment or Toxicity Assessment. Evaluation of toxicity need laboratory animal models or epidemiological studies. This type of assessment often requires observational studies to determine the physical and toxicological properties of a chemical, all of which may take months or years to complete [2,3].

This study aimed to investigate the health effects of fluoride in various types of drinking water in Saveh City, located in Markazi Province, Iran. To evaluate the non-carcinogenic risk of fluoride to human health, we calculated the Estimated Daily Intake (EDI mg/Kg BW per day) and the Hazard Quotient (HQ). Factors such as water consumption, age, and body weight (BW) were taken into account for these calculations. The following equation was utilized for these calculations [4,5]:

(1) $$EDI=\frac{C_{f}\;\times C_{d}}{B_{w}}$$

Where C_d_ is average daily drinking water consumption, C_f_ is concentration of fluoride in drinking water and B_w_ is body weight of consumer. The classification of consumer groups in this study was in 4 categories including infants (0–2 years old), children (2–6 years old), teenagers (6–16 years old) and adults (≥ 16 years old). Body weight and amount of water consumption is presented in Table 2.

**Table 2:** Parameters used to the calculation of fluoride health risk assessment in present data for drinking water [9].

		Specifications of groups				
						
Parameter	Risk exposure factors	Infants	Children	Teenagers	Adults	Unit
Fluoride	C_f_					mg/L
	C_d_	0.08	0.85	2	2.5	L/d
	B_w_	10	15	50	78	Kg
	RfD	0.06	0.06	0.06	0.06	mg Kg^−1^ d^−1^

HQ can be calculated from below equation:

(2) $$HQ=\frac{EDI}{RfD}$$

Where, HQ is hazard quotient and RfD is the reference dose that estimate the daily exposure to the human population that is likely to be without an appreciable risk of deleterious effects during a lifetime. The amount of this parameter for fluoride is 0.06 mg Kg^−1^ d^−1^. HQ value less than one indicates that it is unlikely even for sensitive populations to experience adverse health effects and HQ larger than 1, indicates that the non-carcinogenic risk excesses the acceptable level and adverse health effects are possible [6].

This work confirms the high importance of applied sciences in different fields in nature, and this is shown in a lot of papers published before [7,8].

## Limitations

Not measuring the amount of fluoride in rural areas, due to the lack of financial resources. In these areas, the level of culture, literacy and welfare of the people is lower.

## Ethics Statement

We did not conduct human or animal studies.

## CRediT authorship contribution statement

**Hamid Reza Tashauoei:** Data curation, Visualization. **Mokhtar Mahdavi:** Investigation, Data curation, Writing – original draft. **Amir Hossein Mahvi:** Writing – review & editing. **Ali Fatehizadeh:** Writing – review & editing.

## Declaration of Competing Interest

The authors declare that they have no known competing financial interests or personal relationships that could have appeared to influence the work reported in this paper.

## Data Availability

data for fluoride and Health Risk Assessment (Original data) (Mendeley Data).
